# ING4 Promotes Stemness Enrichment of Human Renal Cell Carcinoma Cells Through Inhibiting DUSP4 Expression to Activate the p38 MAPK/type I IFN-Stimulated Gene Signaling Pathway

**DOI:** 10.3389/fphar.2022.845097

**Published:** 2022-04-14

**Authors:** Yu Tang, Xinyue Yang, Qing Wang, Haoyu Huang, Qinzhi Wang, Min Jiang, Chunluan Yuan, Yefei Huang, Yansu Chen

**Affiliations:** ^1^ Key Laboratory of Human Genetics and Environmental Medicine, School of Public Health, Xuzhou Medical University, Xuzhou, China; ^2^ Key Lab of Environment and Health, Xuzhou Medical University, Xuzhou, China; ^3^ Department of Oncology, First People’s Hospital of Lianyungang, Lianyungang, China

**Keywords:** ING4, p38 MAPK, type I interferon-stimulated genes, cancer stem cells, DUSP4, renal cell carcinoma

## Abstract

Renal cell carcinoma (RCC) recurs frequently due to high metastatic spread, resulting in a high mortality. Cancer stem cells play a critical role in initiating the tumor metastasis. Inhibitor of growth 4 (ING4) is a member of the ING family, but its impact on cancer stem cells in RCC is still unknown. In this study, we found that ING4 significantly promoted the sphere-forming size and number of RCC cells under an ultralow-attachment culture condition *in vitro*, tumor growth and metastasis *in vivo*, and the expression of some stem-like or pluripotent biomarkers CD44, MYC, OCT4, and NANOG, indicating that ING4 increased the stemness enrichment of RCC cells. Mechanistically, the ING4-activated p38 MAPK pathway possibly upregulated the expression of type I IFN-stimulated genes to promote the formation of RCC stem cells. ING4 could inhibit the expression of DUSP4 to activate p38 MAPK. In addition, selective pharmacological p38 MAPK inhibitors could significantly inhibit stemness enrichment only in ING4-overexpressed RCC cells, suggesting that the p38 MAPK inhibitors might be effective in patients with high ING4 expression in RCC tissue. Taken together, our findings proposed that ING4 might serve as a potential therapeutic target for metastatic RCC, particularly RCC stem cells.

## Introduction

Kidney cancer is one of the most common cancers in the world, and renal cell carcinoma (RCC) accounts for 95% of kidney malignancies and is the leading cause of deaths in kidney cancer. Many RCC patients diagnosed incidentally are already advanced, and even after curative nephrectomy, 30% of the RCC patients will relapse and develop metastatic RCC (Sharma et al., 2021). Also, due to high metastatic spread, RCC recurs frequently, ultimately leading to high mortality. The current study shows that the 5-year survival rate for patients with localized RCC is 65%, but it drops to 10–20% once the cancer has metastasized (Znaor et al., 2015). A better understanding of the biology of metastatic RCC may be very helpful for us to improve the clinical outcome of this disease.

Cancer stem cells (CSCs) are characterized in many cancers and contribute to tumorigenesis, recurrence, and metastatic spread (Zhang et al., 2022). In RCC, cell populations with stem-like properties have been isolated, and CSCs are highly enriched in tumors with high pathological stage, venous and lymphatic invasion, and distant metastases (Varna et al., 2015; Fendler et al., 2020). Furthermore, due to the enormous plasticity of tumor cells, CSCs may arise from the dedifferentiation of mature tumor cells (Felipe-Abrio et al., 2019). Therefore, it is essential for us to identify the genetic alterations involved in the regulation of this process.

Inhibitor of growth 4 (ING4) belongs to the conserved ING family (ING1-5). It has been found that a reduced ING4 expression is more prevalent in multiple cancers and associated with poor prognosis, and ING4 deletion increases cell growth, angiogenesis, migration, invasion, and differentiation (Berger et al., 2014; Cui et al., 2015). However, conflicting data have recently been reported for ING4 in the regulation of cellular functions. Moreno et al. (2014) showed that ING4 orchestrates the secretory phenotype in the primary fibroblasts that promotes tumor cell proliferation *in vitro* and *in vivo*. Trinh et al. (2019) demonstrated that ING4 promotes cell proliferation and rRNA synthesis through modulating histone modifications in the rDNA promoters. However, the exact role of ING4 in RCC stemness enrichment has not been illustrated. In this study, we demonstrated that ING4 significantly increased the stemness enrichment of RCC cells. In mechanism, ING4 inhibited DUSP4 expression to activate the p38 MAPK pathway, thereby upregulating the type I IFN-stimulated gene expression.

## Materials and Methods

### Cell Lines

Human RCC cell lines Ketr-3 and 786-O and human embryonic kidney cell line 293T were purchased from the Shanghai Institute of Biochemistry and Cell Biology, Chinese Academy of Sciences (Shanghai, China). 786-O cells were cultured in RPMI-1640 medium supplemented with 10% fetal bovine serum (FBS). Ketr-3 and 293T cells were cultured in DMEM medium with 10% FBS. Cells were grown at 37°C in the presence of 5% CO_2_ in a humidified incubator.

### Lentivirus Infection

The pLenti-GⅢ-CMV-GFP-2A-Puro-HA-ING4 (ING4), pLenti-GⅢ-CMV-GFP-2A-Puro-Vector (Vec), 3 pLenti-U6-sgRNA-SFFV-Cas9-2A-Puro-ING4-target (sgRNA-ING4-1, sgRNA-ING4-2, sgRNA-ING4-3), and pLenti-U6-sgRNA-SFFV-Cas9-2A-Puro-Vector (sgRNA-Vec) (Applied Biological Materials Inc., China) were generated and confirmed by DNA sequencing, and the lentivirus was used to pack these plasmids and infected the RCC cells following the manufacturer’s protocol. These cells were stably selected with 2 μg/ml puromycin for 2 weeks.

### Western Blot and Antibodies

Western blot was carried out as previously reported (Wang et al., 2012). The anti-ING4 (ab108621, 1:1,000; Abcam, United States), anti-CD44 (ab189524, 1:1,000, Abcam, United States), anti-OCT4 (ab200834, 1:1,000, Abcam, United States), anti-MYC (ab32072, 1:1,000, Abcam, United States), anti-NANOG (ab109250, 1:1,000, Abcam, United States), anti-DUSP4 (ab216576, 1:1,000, Abcam, United States), anti-p-p38 MAPK (#4511, 1:1,000, CST, United States), anti-p38 MAPK (#8690, 1:1,000, CST, United States), anti-p-Erk1/2 (#4370, 1:1,000, CST, United States), and anti-Erk1/2 (#4695, 1:1,000, CST, United States) were used for primary antibody incubation at 4°C overnight. The anti-α-tubulin (Cat No.: 11224-1-AP, 1:1,000; Proteintech, China) was used for the protein loading control. Each blot was repeated three times.

### RNA Extraction and Real-Time PCR Analysis

RNA extraction was performed as previously reported (Chen et al., 2014). A real-time PCR was carried out in triplicates on ABI-7500 with HiScript II one step qRT-PCR SYBR Green Kit Q221-01 (Vazyme, China) according to the instruction. GAPDH was used for the normalization of real-time PCR data. The primer sequences are listed as follows:

OCT4-For 5′-GGA​AGG​TAT​TCA​GCC​AAA​CGA​CCA-3′
OCT4-Rev 5′-CTC​ACT​CGG​TTC​TCG​ATA​CTG​GTT-3′
MYC-For 5′-CGA​GGA​GAA​TGT​CAA​GAG​GCG​AAC-3′
MYC-Rev 5′-GCT​TGG​ACG​GAC​AGG​ATG​TAT​GC-3′
CD44-For 5′-ACA​AGC​ACA​ATC​CAG​GCA​ACT​CC-3′
CD44-Rev 5′-TGG​TGT​TGT​CCT​TCC​TTG​CAT​TGG-3′
NANOG-For 5′-GCC​TCC​AGC​AGA​TGC​AAG​AAC​TC-3′
NANOG-Rev 5′-CCA​GGT​CTG​GTT​GCT​CCA​CAT​TG-3′
IFITM1-For 5′-GAT​CAA​CAT​CCA​CAG​CGA​GAC​CTC-3′
IFITM1-Rev 5′- GCC​CAG​ACA​GCA​CCA​GTT​CAA​G-3′
IFITM2-For 5′- AGA​CCT​CCG​TGC​CTG​ACC​ATG-3′
IFITM2-Rev 5′- CGT​CGC​CAA​CCA​TCT​TCC​TGT​C-3′
MX2-For 5′- GAG​GCA​GCA​GAC​GAT​CAA​CTT​GG-3′
MX2-Rev 5′- CCG​ATG​GTC​CTG​TCC​CCT​TCC-3′
OAS2-For 5′- TCC​GAC​AAT​CAA​CAG​CCA​AGA​TCC-3′
OAS2-Rev 5′- GCA​TCA​GAG​CCA​GTC​TTC​AGA​GC-3′
DUSP4-For 5′-TGT​GCT​GCG​GAG​GCT​GCT​AG-3′
DUSP4-Rev: 5′-GCT​GAA​GAC​GAA​CTG​CGA​GGT​G-3′
GAPDH-For 5′-AAG​GTC​GGA​GTC​AAC​GGA​TTT​G-3′
GAPDH-Rev 5′-CCA​TGG​GTG​GAA​TCA​TAT​TGG​AA-3′

### Sphere Formation Assay

RCC cells were seeded in low-adherent 24-well culture plates (Corning, NY, United States) at 1 × 10^3^ cells per well, and incubated in DMEM/F12 (Gibco) containing 20 μl/ml B27 (Gibco), 20 ng/ml epidermal growth factor (EGF) (Gibco), 20 ng/ml of basic fibroblast growth factor (bFGF) (Gibco), 5 μg/ml insulin (Sigma), and 1% penicillin–streptomycin (Beyotime Biotechnology, China) under a serum-free condition. After 7 days of incubation at 37°C in a 5% CO_2_ incubator, photographs were taken under a microscope and the number of cell spheres was counted in three separates in 100× fields. The cell sphere was defined when it reached a diameter ≥50 μm, and the differences in cell sphere number and size was determined mainly by comparing the number of cell spheres with diameters ≥50 μm or ≥100 μm between groups.

### Tumor-Bearing and Metastasis Experiments

All animal studies were approved by the Animal Ethics Committee of Xuzhou Medical University (ethical number: 202009A034). For *in vivo* tumor growth and metastasis experiments, 20 six-week-old male BALB/c nude mice (Shanghai Slac Laboratory Animal Co. Ltd., China) were randomly divided into four groups (5 mice/group). 1 × 10^6^ ING4 knockout or vector control 786-O cells were suspended into 200 μl PBS and subcutaneously or intravenously injected into the armpit of right forelimb or the tail vein, respectively. The tumor growth assay lasted for 6 weeks, and the tumor metastasis model lasted for 8 weeks.

### Immunohistochemistry and Assessment of IHC

The slides were dewaxed three times with xylene for 15 min each, washed in 100, 85, and 75% ethanol for 5 min each and distilled water, and then put into boiling sodium citrate (pH 6.0) for 2 min to retrieve the antigen and washed with PBS (pH 7.4) three times for 5 min each. The endogenous peroxidase activity of the tissue was blocked by incubation in 3% hydrogen peroxide for 30 min and washed with PBS (pH 7.4) three times for 5 min each. The sections were blocked in 3% BSA for 30 min, and then incubated with polyclonal rabbit anti-CD44 antibody (GB14037, 1:300 dilution; Servicebio, China), anti-MYC antibody (GB13076, 1:300 dilution; Servicebio, China), anti-NANOG antibody (GB11331, 1:500 dilution; Servicebio, China), and anti-ING4 (HPA057338, 1:5,000 dilution; Sigma-Aldrich, United States) at 4°C overnight and washed with PBS (pH 7.4) three times for 5 min each. The sections were then incubated for 50 min each with a HPR-labeled secondary antibody (1:200 dilution; Servicebio, China) and washed with PBS (pH 7.4) three times for 5 min each. The sections were developed using DAB (GB1211, Servicebio, China) and counterstained with hematoxylin. The slides were then dehydrated according to the standard procedures and sealed with coverslips.

Immunohistochemistry assessment was performed as the previous study (Wang et al., 2012). The staining of ING4, MYC, CD44, and NANOG in the tissue was scored by applying a semi-quantitative immunoreactivity score (IRS). Category A and category B documented the intensity of immuno-staining as 0–3 (0, negative; 1, weak; 2, moderate; and 3, strong) and the percentage of the immunoreactive cells as 1–4 (1, 0–25%; 2, 26–50%; 3, 51–75%; and 4, 76–100%). The IRS of tumor tissue ranged from 0 to 12 after the multiplication of category A and category B.

### Transcriptome Sequencing and Quantitative Analysis

Human transcriptome sequencing (Vazyme Biotech, China) was used to analyze the gene expression collected in triplicates from the ING4-overexpressed and corresponding control 786-O cells. The quantitative analysis of gene expression was performed using Cufflinks (cufflinks-2.2.1). The gene expression was calculated as the FPKM (expected number of Fragments Per Kilobase of transcript sequence per Millions base pairs sequenced). Gene expression differences between the samples were calculated by the Cuffdiff analysis in Cufflinks. Volcano plots were used to show the differentially expressed genes with a criterion of |log2Ratio| ≥ 0.585 and *p* value ≤0.05. Gene Ontology (GO) function enrichment analysis was used to give biological functions, and a smaller q-value indicated a higher enrichment of differential genes. The vertical coordinate was the enriched GO terms, and the horizontal coordinate was the number of significantly different genes in that term.

### CCK-8 Assay

Human embryonic kidney cells 293T were seeded directly into 96-well culture plates at 5×10^3^ cells/well and cultured in 100 μl complete DMEM medium with 5 μM SB203580 and 1 μM BIRB0796 for 4, 24, 48, and 72 h. At the exact time, 10 μl CCK-8 solution (#40203ES76, Yeasen Biotechnology (Shanghai) Co., Ltd., China) was added to each well and incubated at 37°C for 1.5 h. By using an ELX-800 spectrometer reader, the absorbance was measured at 450 nm.

### Statistical Analysis

Student’s t-test or ANOVA was used to evaluate the significance of quantitative data between the two groups or among ≥3 groups, and then Scheffe’s test was used to identify the significant differences between the two groups when ≥3 groups were present. All the statistical analyses were performed by STATA statistical software (version 14; StataCorp, College Station, TX). *p* value ≤0.05 was deemed statistically significant, and all tests were two-sided.

## Results

### ING4 Promoted Stemness Enrichment of RCC Cells

**FIGURE 1:**
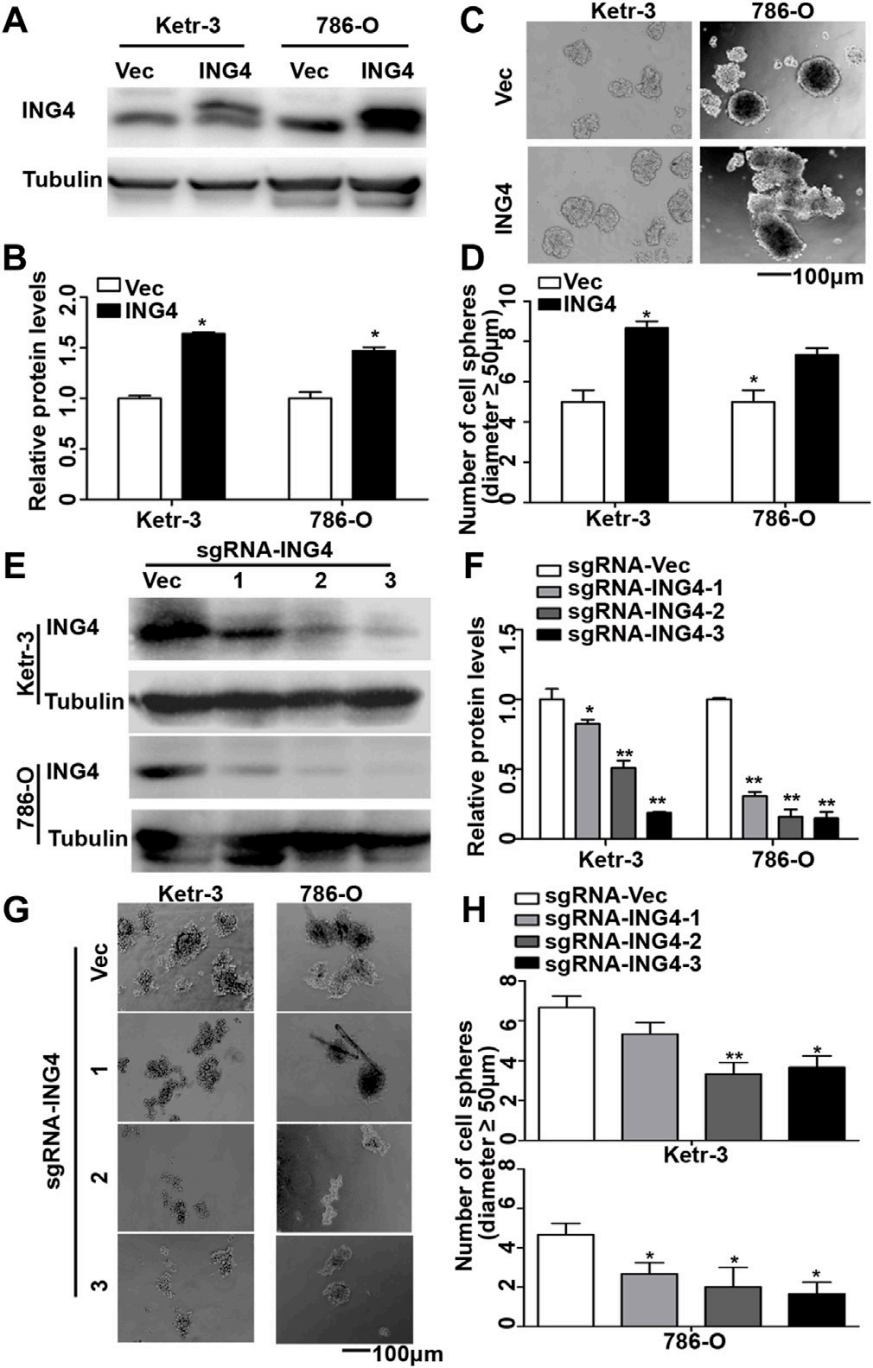
ING4 promoted the formation of RCC cell sphere. **(A–B)** Lentivirus-mediated ING4 and its vector (Vec) was stably overexpressed in the Ketr-3 and 786-O cells and relative protein expression of ING4 was normalized to that of the respective α-tubulin. **(C–D)** Photographs and number of cell spheres in ING4 overexpression (ING4) and control (Vec) Ketr-3 and 786-O cells (n = 3). **(E–F)** Three lentivirus-mediated ING4-targeted sgRNAs (sgRNA-ING4) and vector (sgRNA-Vec) were used to stably knockdown the ING4 expression in Ketr-3 and 786-O cells and relative protein expression of ING4 was normalized to that of the respective α-tubulin. **(G–H)** Photographs and number of cell spheres in ING4 knockdown and control Ketr-3 and 786-O cells (n = 3). Image magnification, ×100; scale bar, 100 μm; data are presented as mean ± standard deviation. *p < 0.05, **p < 0.001.

Tumor metastasis causes approximately 90% of tumor-related deaths. Notably, at least 80% of the metastases are initiated in a clinically latent stage (Hosseini et al., 2016). CSCs initiate tumor metastasis and display the highest metastatic potential (Cole et al., 2020). To investigate the function of ING4 in the formation of RCC CSCs, we at first stably overexpressed ING4 with lentivirus in two RCC cell lines, KETR-3 and 786-O (Figures 1A,B), and then performed a sphere formation assay under an ultralow-attachment culture condition. Our data showed that ING4 overexpression significantly increased the sphere-forming size and number in both RCC cell lines compared to the respective controls (Supplementary Figure S1A and Figures 1C,D). Meanwhile, we further produced three lentivirus-mediated sgRNAs to stably knock down ING4 expression in these two RCC cells (Figures 1E,F), and under ultralow-attachment culture conditions, we found that ING4 knockdown significantly reduced the sphere-forming size and number in RCC cells compared to the respective controls (Supplementary Figure S1B and Figures 1G,H).

**FIGURE 2:**
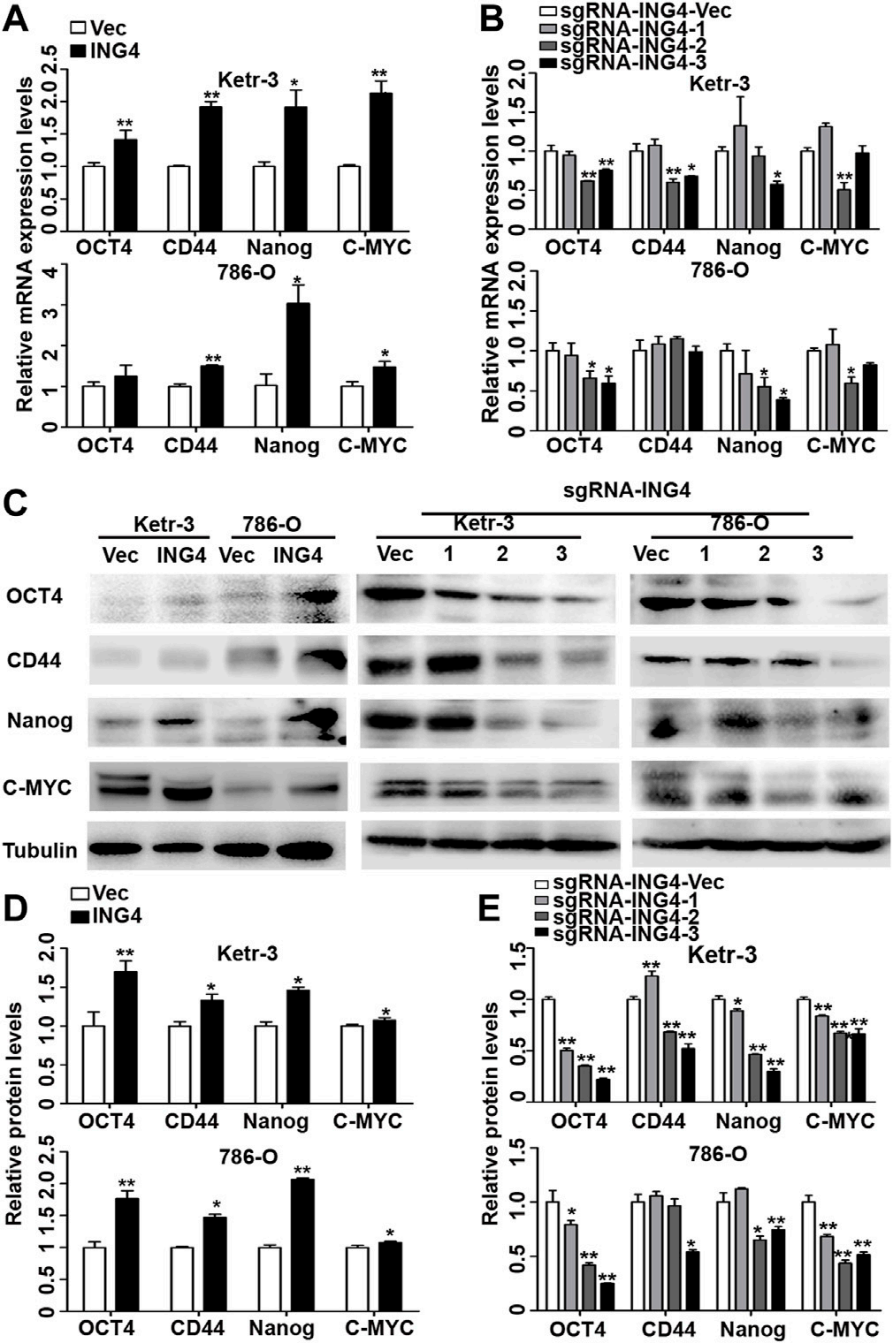
ING4 increased the expression of stem cell markers in RCC cells. **(A–B)** Real-time PCR was used to test the relative mRNA expression of stem cell markers OCT4, CD44, NANOG, and MYC in ING4-overexpressed or knockdown Ketr-3 and 786-O cells compared to the respective vector control. **(C–E)** Western blot was performed to test the protein expression levels of OCT4, CD44, NANOG, and MYC in ING4-overexpressed or knockdown Ketr-3 and 786-O cells and relative protein expression was normalized to that of the respective α-tubulin. Data are presented as mean ± standard deviation. *p < 0.05, **p < 0.001.

Furthermore, we tested the expression levels of some common RCC CSC biomarkers including OCT4, CD44, NANOG, and MYC (Hu et al., 2017; Qian et al., 2018; Rasti et al., 2018), and found that the ING4 overexpression significantly promoted the mRNA expression levels of almost all stem cell markers in Ketr-3 and 786-O cells compared to the respective controls (Figure 2A). In addition, compared to the respective controls, sg-ING4-2 or sg-ING4-3 dramatically suppressed the mRNA expression levels of OCT4, CD44, MYC, and NANOG in Ketr-3 cells, and decreased or showed a decreasing trend in the mRNA expression of OCT4, MYC, and NANOG in 786-O cells (Figure 2B). Next, western blot showed that the expression levels of these stem cell markers increased dramatically after ING4 overexpression and decreased after ING4 knockdown compared to the respective vector control (Figures 2C–E).

### ING4 Promoted Tumor Growth and Lung Metastasis *In Vivo*

**FIGURE 3:**
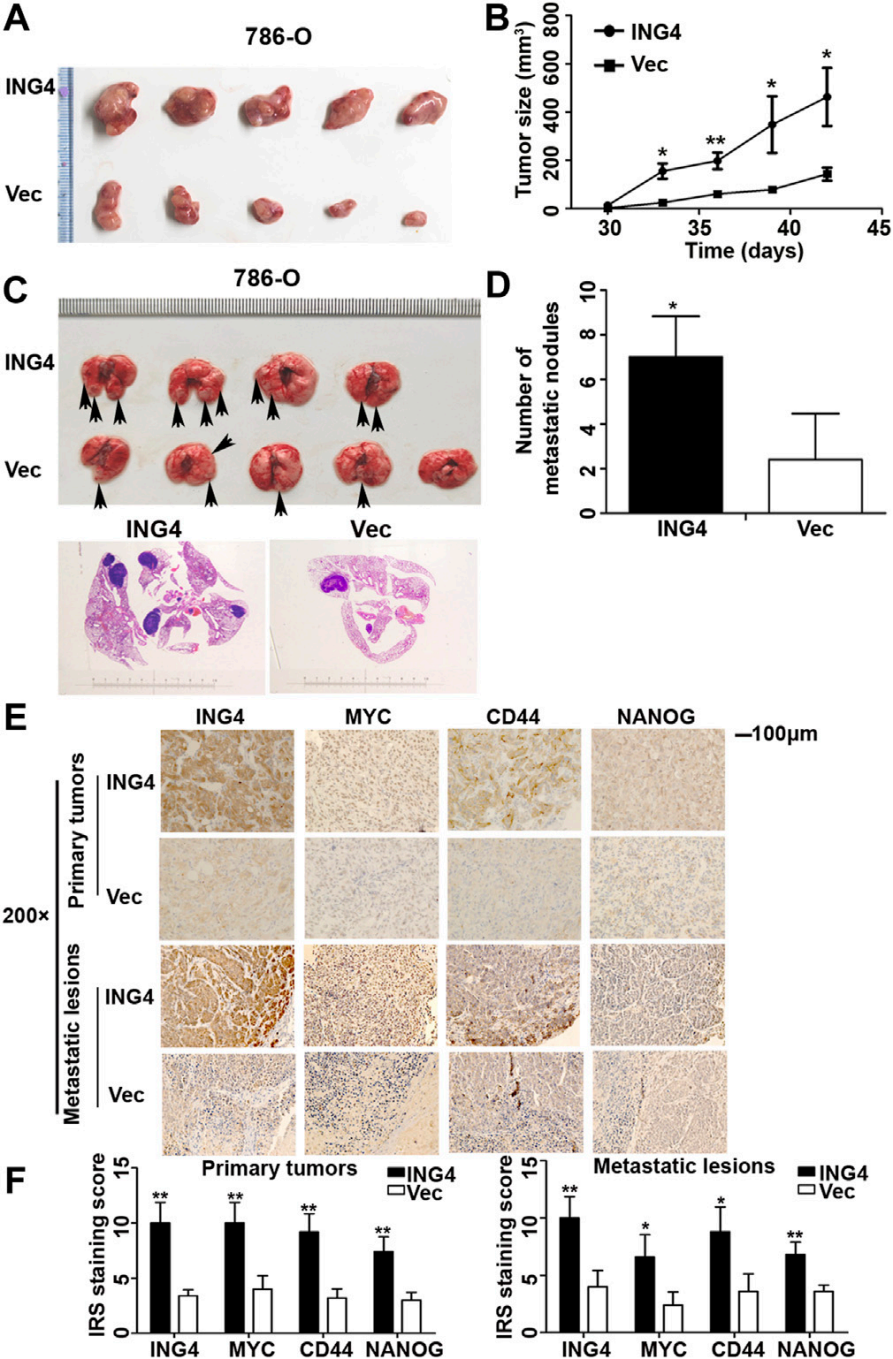
ING4-promoted tumor growth and lung metastasis *in vivo*. **(A–B)** The picture of xenograft tumors and the size change of these tumors arising from ING4-overexpressed (ING4) and vector control (Vec) 786-O cells from 30 to 42 days. **(C)** The representative lung images of mice and HE staining of the lung in the stable ING4-overexpressed group (ING4) and control 786-O group (Vec). **(D)** The number of lung metastatic nodules was counted. **(E–F)** The representative IHC images and IRS staining scores of ING4, MYC, CD44, and NANOG in the primary tumors collected from the tumor-bearing experiment and metastatic lesions collected from the tail vein metastasis model (n = 5). IHC image magnification, ×200; scale bar, 100μm; data were presented as mean ± standard deviation, *p < 0.05, **p < 0.001.

CSCs exhibit the uncontrollable ability for cellular growth, and essence and origin of tumor metastasis is the transfer and homing of CSCs (Parmiani, 2016). Here, we explored whether ING4 could promote tumor growth and metastasis *in vivo*. The tumor-bearing experiment showed that tumors arising from ING4 stably overexpressed 786-O cells tended to be much larger and grew faster than those developed from the control cells (Figures 3A,B). In the tail vein metastasis model, ING4 stably overexpressed or control 786-O cells were injected intravenously into BALB/C nude mice (five mice/group). During the experiment, one mouse in the ING4 overexpression group died due to lung metastasis. Two months later, all mice were sacrificed and visual inspection and HE staining showed the presence of metastatic lesions in the lungs, which were more frequent in the ING4 overexpression group compared to the control group (Figures 3C,D). Then, the protein expression levels of ING4 and some stem-like or pluripotent biomarkers CD44, MYC, and NANOG were detected in the primary tumors and metastatic lesions collected from *in vivo* experiments. Our results showed that the expression of ING4, MYC, CD44, and NANOG were significantly higher in tumors with ING4 overexpression than with the control (Figures 3E,F).

### Type I Interferon-Stimulated Genes Played an Important Role in the ING4-Promoted Stemness Enrichment of RCC Cells

**FIGURE 4:**
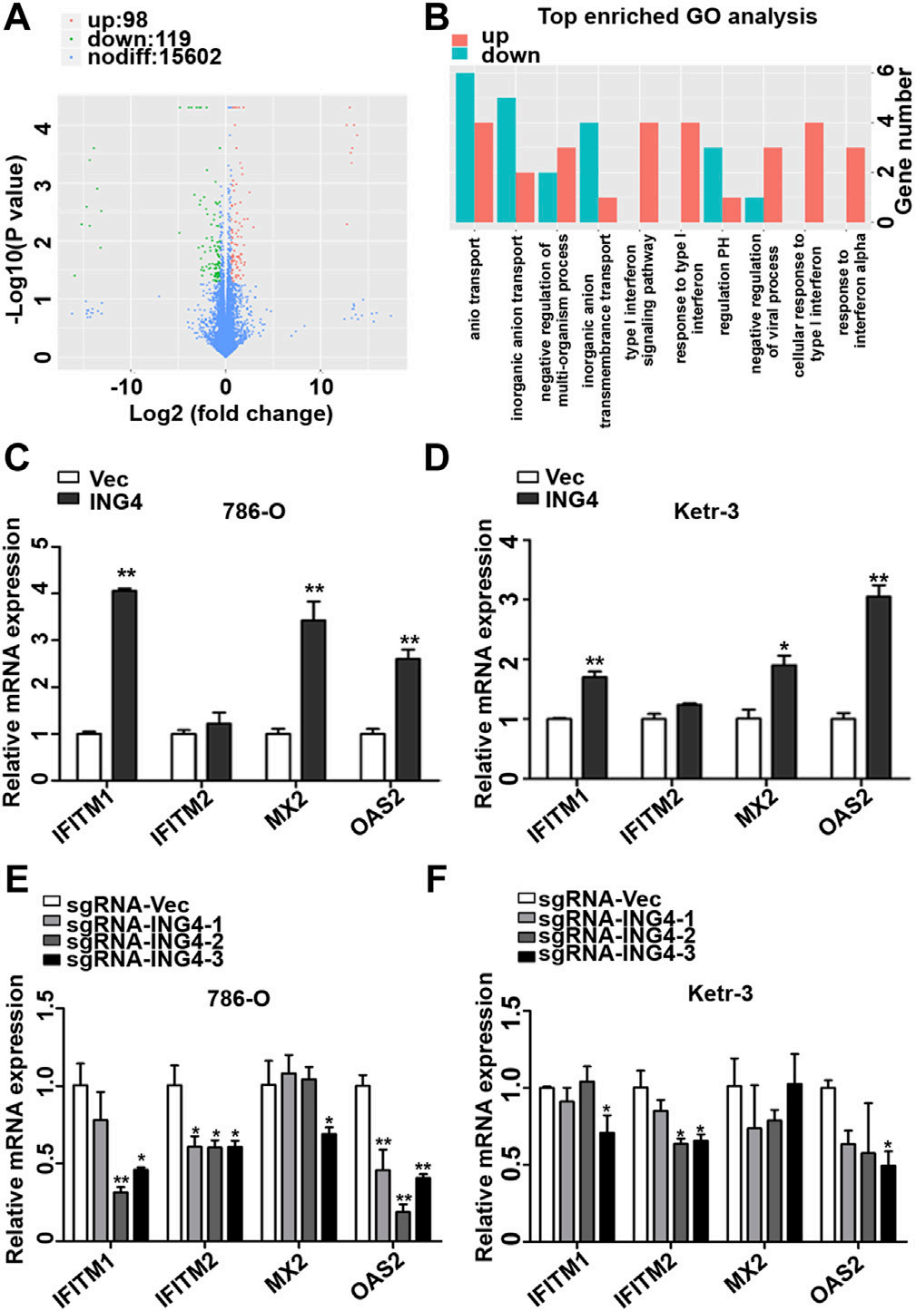
ING4-increased ISG expression. **(A)** The volcano plot was used to indicate the significantly differential gene expressions in 786-O cells. **(B)** GO analysis was used to identify the functional enrichments in the biological process, the vertical coordinate was the enriched GO term, and the horizontal coordinate was the number of significantly different genes in that term. **(C–D)** The real-time PCR analyzed the mRNA expression levels of ISGs, including OAS2, MX2, IFITM1, and IFITM2 in ING4-overexpressed (ING4) 786-O and Ketr-3 cells compared to the respective vector controls (Vec). **(E–F)** The relative mRNA expression levels of ISGs in ING4 knockdown (sgRNA-ING4) Ketr-3 and 786-O cells were compared to the respective vector control (sgRNA-Vec). Data are presented as mean ± standard deviation. *p < 0.05, **p < 0.001.

In order to further search for possible downstream targets of ING4 in the promotion of RCC CSCs formation, we performed transcriptome sequencing in the ING4 overexpressed and vector control 786-O cells. The volcano plot showed that ING4 upregulated 98 genes and downregulated 119 genes (Figure 4A). GO analysis was used to identify the functional enrichments and showed that the upregulated type I IFN signaling pathway, response to type I IFN, cellular response to type I IFN, and response to IFN-α were enriched in the top ten GO terms with low false discovery rate values in the biological process (Figure 4B). IFN-stimulated genes (ISGs) IFITM1, IFITM2, MX2, and OAS2 coenriched in these GO terms were significantly upregulated in 786-O cells with stable ING4 overexpression compared to vector controls (Supplementary Table S1). And the protein interaction network also showed that IFITM1, IFITM2, MX2, and OAS2 were associated with many proteins and formed a core of protein interactions (Supplementary Figure S2).

Accumulative evidences demonstrate that the type I IFN signaling pathway and its driving ISGs are cancer stemness drivers in various cancers (Martin-Hijano and Sainz, 2020). Therefore, we sought to explore the link between the type I IFN signaling pathway and ING4-induced CSCs formation. We conducted a real-time PCR of ISGs including IFITM1, IFITM2, MX2, and OAS2, discovered from GO analysis and found that IFITM1, MX2, and OAS2 were significantly and substantially upregulated and IFITM2 had a slight upregulation trend in ING4 overexpressed 786-O and Ketr-3 cells compared to their respective vector controls (Figures 4C,D). To validate the relationship of ING4 with IFITM1, IFITM2, MX2, and OAS2, we tested their mRNA expression in ING4 knockdown and vector control RCC cells, and found that the mRNA expression levels of these genes were significantly suppressed or showed a decreasing trend after ING4 knockdown (Figures 4E,F).

### ING4 Promoted Stemness Enrichment of RCC Cells Through Activating the p38 MAPK/type I IFN-Stimulated Gene Signaling Pathway

**FIGURE 5:**
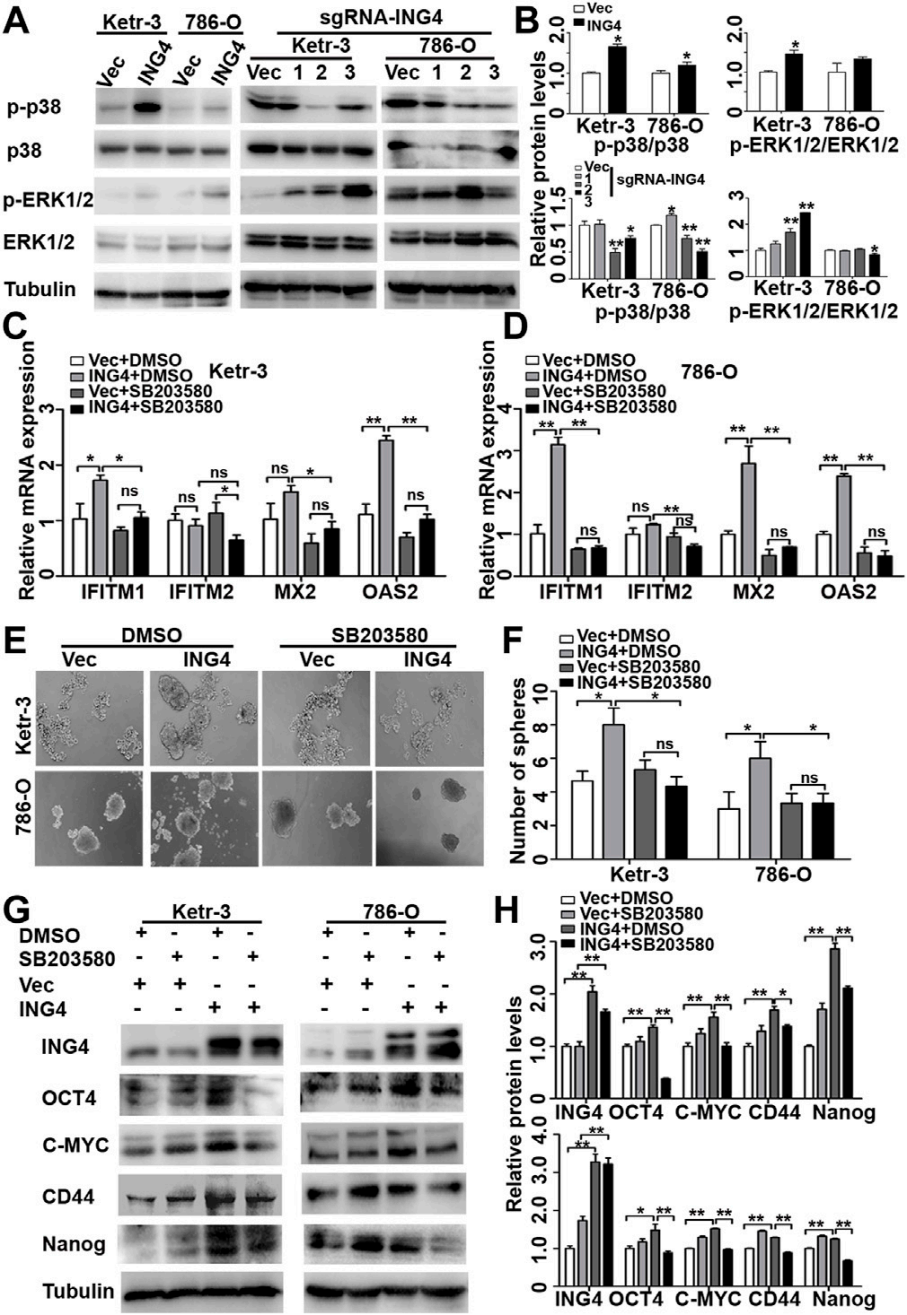
ING4 promoted stemness enrichment of RCC cells through the activation of the p38 MAPK/type I IFN-stimulated gene signaling pathway. **(A–B)** Western blot showed levels of p38 MAPK and Erk1/2 activation in ING4-overexpressed (ING4) or knockdown (sgRNA-ING4) Ketr-3 and 786-O cells, and the relative protein expression of p-p38 or p-Erk1/2 was normalized to that of the respective p38 or Erk1/2. **(C–D)** Real-time PCR analyzed the mRNA expression levels of ISGs including OAS2, MX2, IFITM1, and IFITM2 in 786-O and Ketr-3 cells with ING4 overexpression (ING4) or vector control (Vec) after 0.1%DSMO or 5 μM SB203580 treatment for 24 h. **(E–F)** Photographs and number of cell spheres in 786-O and Ketr-3 cells with ING4 overexpression (ING4) or vector control (Vec) after 0.1%DSMO or 5 μM SB203580 pretreatment for 24 h (n = 3). **(G–H)** Western blot tested the protein expression levels of stem cell markers OCT4, CD44, MYC, and NANOG in 786-O and Ketr-3 cells with ING4 overexpression (ING4) or vector control (Vec) after 0.1%DSMO or 5 μM SB203580 treatment for 24 h and relative protein expression was normalized to that of the respective α-tubulin. Note: images magnification, ×100; Scale bar, 100 μm; Data are presented as mean ± standard deviation. *p < 0.05, **p < 0.001, ns: no significance.

The activation of MAPK has been reported to play an important role in type I IFN signaling pathway for CSCs formation (Patel et al., 2016; Fang et al., 2017; Lu et al., 2018; Kirk et al., 2021), and ING4 can regulate the Erk1/2 and p38 MAPK activity (Zhang et al., 2007). We examined the activation status of Erk1/2 and p38 MAPK in ING4-overexpressed or knockdown Ketr-3 and 786-O cells and found that the phosphorylation level of p38 MAPK was significantly increased in the ING4 overexpression group and decreased in the sg-ING4-2 and sg-ING4-3 groups compared to the respective control groups. However, there was no consistent change in the phosphorylation of Erk1/2 (Figures 5A,B).

Since studies have shown that the p38 MAPK pathway plays a critical role in type I IFN-induced transcriptional activation of hundreds of ISGs (Saleiro et al., 2015), we examined the possibility that the effect of ING4 on ISG transcription are mediated by p38 MAPK. We found that a selective pharmacological p38 MAPK inhibitor SB203580 significantly abrogated ING4-mediated upregulation IFITM1, MX2, and OAS2 in Ketr-3 and 786-O cells (Figures 5C,D). Then, we examined whether p38 MAPK was responsible for the stemness of RCC cells promoted by ING4. We pretreated Ketr-3 and 786-O cells with 5 μM SB203580 or 1 μM BIRB0796 for 24 h, and then examined the cell sphere formation, and found that SB203580 and BIRB0796 showed no significant inhibitory effect on vector cells, but almost completely impaired ING4 overexpression-promoted number and size of cell spheres formed by RCC cells (Figures 5E,F, Supplementary Figures S3, S4). Similarly, we observed that only in ING4-overexpressed Ketr-3 and 786-O cells, SB203580 exposure resulted in a significant downregulation of stemness markers OCT4, MYC, CD44, and NANOG compared to 0.1% DMSO exposure (Figures 5G,H). These data suggested that the activation of p38 MAPK was required for the ING4-mediated CSC formation of RCC cells. In addition, the p38 inhibitors showed no significant inhibitory effect on the viability of non-cancer-derived human embryonic kidney cells 293T (Supplementary Figure S5).

### ING4 Inhibited DUSP4 Expression to Regulate p38 MAPK Activity

**FIGURE 6:**
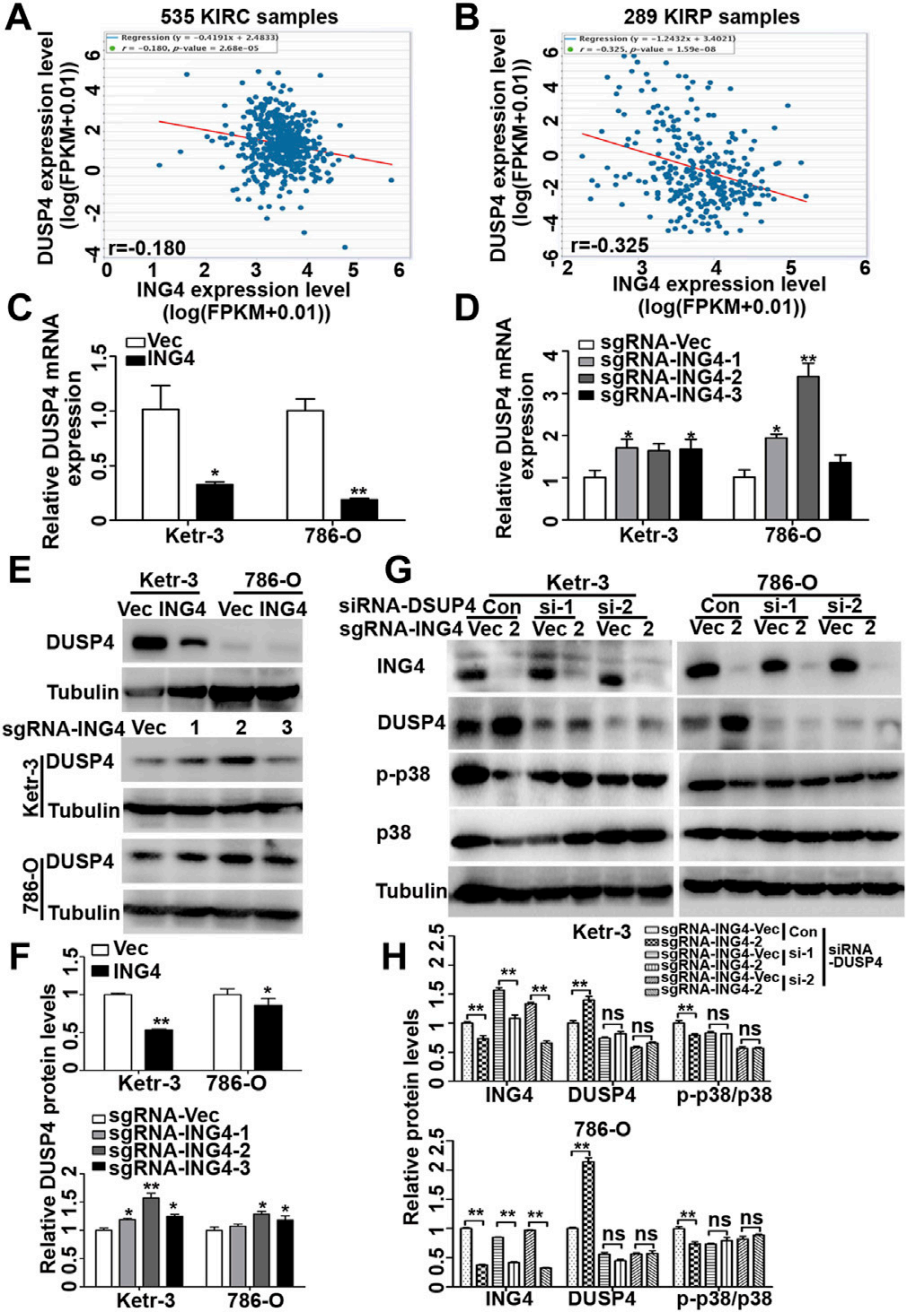
ING4 inhibited the DUSP4 expression to regulate the p38 MAPK activity. **(A–B)** The mRNA correlation of ING4 with DUSP4 in TCGA-KIRC and TCGA-KIRP from the starBase v3.0 project. **(C–D)** Real-time PCR was used to test the relative mRNA expression levels of DUSP4 in ING4-overexpressed (ING4) or knockdown (sgRNA-ING4) Ketr-3 and 786-O cells compared to the respective vector controls. **(E–F)** Western blot was used to test the protein expression of DUSP4 in ING4 overexpression (ING4) or knockdown (sgRNA-ING4) or their respective vectors Ketr-3 and 786-O cells and relative protein expression was normalized to that of respective α-tubulin. **(G–H)** Western blot was used to test the expression of ING4, DUSP4, p-p38, and p38 in ING4 knockdown (sgRNA-ING4-2) or vector control (sgRNA-Vec) Ketr-3 and 786-O cells after transfection with DUSP4 (si-DUSP4) or control siRNA (si-Con), and the relative protein expression of ING4 was normalized to that of the respective α-tubulin. Data are presented as mean ± standard deviation. *p < 0.05, **p < 0.001.

Dual-specificity phosphatases (DUSPs), also known as mitogen-activated protein kinase phosphatases (MKPs) and the most powerful regulators of intensity and duration of MAPK signaling, including DUSP1, DUSP2, DUSP4, DUSP6, DUSP7, DUSP8, DUSP9, DUSP10, DUSP14, DUSP16, and DUSP26, have been validated as the major negative regulators of p38 MAPK activation (Patterson et al., 2009; Chen et al., 2019). Therefore, here, we further investigated whether DUSPs played an important role in ING4-acitivated p38 MAPK. Using TCGA-Kidney Renal Clear Cell Carcinoma (KIRC) and TCGA-Kidney Renal Papillary Cell Carcinoma (KIRP) datasets publicly available in the ENCORI Pan-Cancer Analysis Platform (http://starbase.sysu.edu.cn/panCancer.php), we found that in both TCGA-RCC datasets, only the DUSP4 expression had a consistent and significantly negative relationship with ING4 (Figures 6A,B).

**FIGURE 7:**
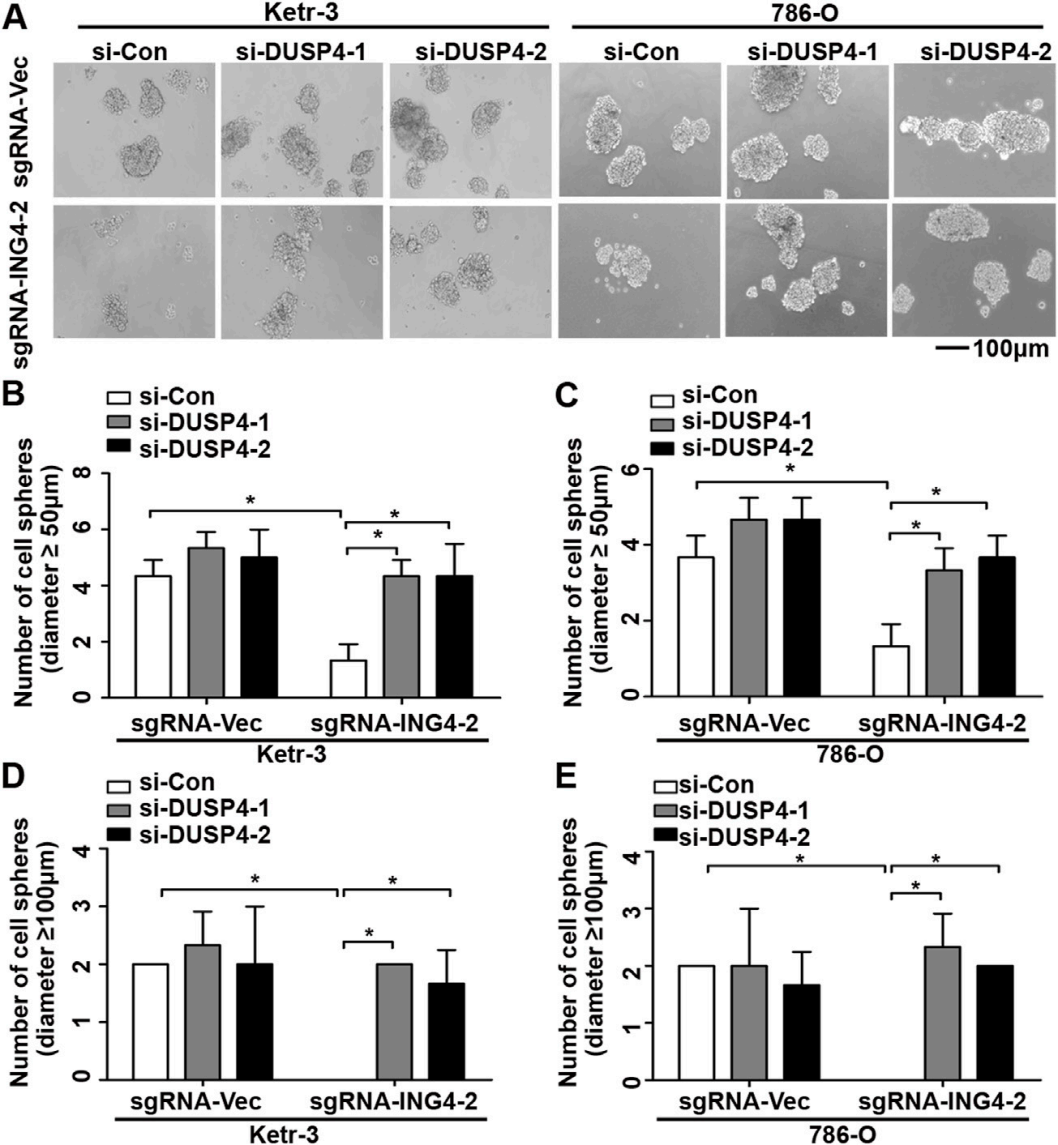
Inhibition of DUSP4 expression significantly reversed the reduction of cell sphere formation after ING4 knockdown. **(A)** Photographs of cell spheres in ING4 knockdown (sgRNA-ING4-2) or vector control (sgRNA-Vec) Ketr-3 and 786-O cells after transfection with DUSP4 (si-DUSP4) or control siRNA (si-Con) (n = 3). **(B–E)** The number of cell spheres with diameters ≥50 μm or ≥100 μm. Image magnification, ×100; scale bar, 100μm; data are presented as mean ± standard deviation. *p < 0.05.

Next, using real-time PCR, we found that in Ketr-3 and 786-O cells, the mRNA expression levels of DUSP4 were greatly suppressed by ING4 overexpression and significantly promoted or showed a slight upregulation trend by ING4 knockdown compared to the respective controls (Figures 6C,D); consistent with the PCR results, western blot showed similar changes in the protein expression of DUSP4 (Figures 6E,F). Moreover, the p38 MAPK activity inhibited by the ING4 knockdown could be greatly restored when two DUSP4-specific siRNAs were transfected into ING4 knockdown Ketr-3 and 786-O cells (Figures 6G,H). Furthermore, sphere formation assay was performed and showed that DUSP4 knockdown significantly reversed the reduction in the number and size of cell sphere formation caused by ING4 knockdown in Ketr-3 and 786-O cells (Figure 7). These data suggested that DUSP4/p38 MAPK pathway might play an important role in ING4-promoted stemness enrichment.

## Discussion

Metastasis is the most important step in the progression of cancer. Though metastasis arises in the late stage of cancer, studies have shown that at least 80% of the metastases are derived from the early cancer cell dissemination in a clinically latent stage (Hosseini et al., 2016). Therefore, it is pivotal to understand the initiation of metastasis. CSCs have been reported to initiate tumor metastasis (Zhang et al., 2022). The stem cell biomarker CD44 and pluripotent transcription factors NANOG, MYC, and OCT4 have now been validated to contribute to the maintenance of CSCs and are widely used to identify CSCs. Siddique et al. (2015) showed that NANOG regulates the self-renewal of liver tumor-initiating cells *via* NUMB-p53 signaling axis; Akita et al. (2014) showed that MYC induces the self-renewal capacity of liver cancer cells in a p53-dependent manner; and Koo et al. (2015) showed that OCT4 dedifferentiates differentiated head and neck squamous carcinoma cells to CSCs-like cells. In this study, we found that ING4 significantly promoted the sphere-forming ability and the expression levels of stem cell biomarker CD44 and pluripotent transcription factors NANOG, MYC, and OCT4 in RCC cells. Moreover, CSCs are characterized by unlimited tumor growth and are responsible for tumor metastasis (Parmiani, 2016). Our data showed that ING4 significantly accelerated tumor growth and metastasis *in vivo*, and combined with the IHC results of tumor tissues from *in vivo* experiments, we also validated the regulatory relationship between ING4 and pluripotent or stem-like biomarkers. It has been shown that ING4 is the homolog of ING5 and possesses similar function to ING5 which has been indicated to play an important role in maintaining self-renewal of embryonic stem cell lines and cancer stem cells (Guerillon et al., 2014; Wang et al., 2018). In addition, Shiseki et al. (2003) noted that the ING4 overexpression significantly reduces the colony formation of the wild p53 cell lines, but has no significant effect on colony formation of the inactivated or mutated p53 cell lines, suggesting that ING4 exerts its tumor-suppressive function depending on the status of p53. It is well known that p53, referred to as the “guardian of the human genome,” is inactivated directly by mutation in 50% of human cancers (ranging from about 1 to 85% depending on the type of cancer), and almost all cancers exhibit dysfunction of the p53 pathway (Miller et al., 2020). In this study, we searched the p53 status of cell lines we used in the Expasy (https://web.expasy.org/cellosaurus/), and found that 786-O was a p53-mutated RCC cell line. Thus, in combination with these reports, we proposed that p53 mutated or inactivated status might play an important role in the cancer stem cell properties promoted by ING4 in RCC cells, at least in the case of 786-O cells.

In order to further seek the possible downstream targets in the ING4-mediated activation of RCC CSCs formation, we performed transcriptome sequencing and found that ING4 overexpression resulted in upregulated type I IFN-related responses being enriched at low false discovery rate values and type I IFN-driven ISGs, including IFITM1, IFITM2, MX2, and OAS2, clearly formed a protein interaction core. Though some studies show that the activation of the type I IFN pathway inhibits the stem-like capacities of CSCs (Doherty et al., 2017; Martin-Hijano and Sainz, 2020), a growing number of studies suggest that type I IFN (specially IFN-α) is considered as “awakening” agents for dormant CSCs, and the activated type I IFN signaling pathway actively promotes the downstream ISGs expression, induces self-renewal capacity, tumorigenic potential, expression of stem-like markers, and activates EMT in many kinds of cancers (Martin-Hijano and Sainz, 2020). In this study, the real-time PCR was used to validate the findings in transcriptome sequencing, which revealed that ING4 significantly and greatly increased the mRNA expression levels of ISGs, including OAS2, MX2, IFITM1, and IFITM2. These data suggested that type I IFN-stimulated genes played an important role in the ING4-promoted stemness enrichment of RCC cells.

Previous evidence suggests that the p38 MAPK pathway appears to serve as an important pathway, necessary for the optimal transcription of ISGs (Saleiro et al., 2015), and ING4 has been reported to regulate Erk1/2 and p38 MAPK activity (Zhang et al., 2007). In our study, we found that ING4 positively regulated the p38 MAPK activity and inhibition of p38 MAPK activity almost completely abrogated the ING4-mediated upregulation of ISGs. Indeed, the functions of p38 MAPK in cancer have been widely investigated. Evidences have indicated that the stage of tumor development can strongly govern the function of p38 MAPK, with a low p38 MAPK activity generally favoring malignancy at early stages and p38 MAPK being highly activated at the advanced stages of tumorigenesis, involving migration to adjacent organs (Martinez-Limon et al., 2020; Pua et al., 2022). In addition, the role of p38 MAPK in CSC regulation has been validated. p38 MAPK appears to promote the survival and mediates drug resistance in CSCs (Chen et al., 2012; Lin et al., 2012), and inhibition of p38 MAPK reduces the formation of CSCs and promotes the sensitization of CSCs to the chemotherapeutic agents (Roy et al., 2018; Lepore Signorile et al., 2021). Here, we found that inhibiting the p38 MAPK activation could significantly inhibit the ING4-promoted stemness enrichment of the RCC cells and the expression of CSCs biomarker, but there was no significant toxic inhibitory effect on the viability of non-cancer-derived kidney cells, indicating that p38 MAPK inhibitors might be effective for the patients with high ING4 expression in the RCC tissues. Collectively, these results suggested a mechanism by which ING4 might promote the stemness of the RCC cells by activating the p38 MAPK/type I IFN-stimulated genes signaling pathway.

Although MAPK signaling can be modulated at different levels, MKPs exert a tight control, inactivating MAPK proteins ERK, p38, and JNK with a 10–100 stronger potency than the upstream kinases, indicating that MKPs are the most powerful regulators of MAPK signaling intensity and duration (Perez-Sen et al., 2019). Using the TCGA datasets, we found that only DUSP4, a p38 MAPK-related MKP, was significantly and negatively associated with the ING4 expression. Since ING4 or ING5 has been validated to bind to HBO1 complexes to mediate the anchorage of these complexes on chromatin to regulate chromatin modification and gene transcription (Saksouk et al., 2009), here we sought to explore the regulatory relationship of ING4 with DUSP4, and found that ING4 negatively regulated DUSP4 mRNA and protein expression in RCC cells. Moreover, the inhibition of DUSP4 expression significantly restored the ING4 knockdown-mediated suppression of p38 MAPK activity and reversed the ING4 knockdown-reduced cell sphere formation. These data revealed that ING4 could inhibit DUSP4 to regulate the p38 MAPK activity and stemness enrichment of RCC cells. In the present study, we also found that the level of p-p38 was not significantly upregulated but somewhat downregulated after DUSP4 knockdown alone. It has been reported that the induction and transcription of DUSPs are dependent on the activation of MAPK, and once expressed, DUSPs are able to bind directly to the activation loop of MAPK, resulting in MAPK dephosphorylation and inactivation, which provides a negative feedback loop to dampen the extent and duration of MAPK signaling (Ferguson et al., 2016; Liu and Molkentin, 2016). Here, we proposed that it might be that DUSP4 knockdown-activated other MAPK could induce compensatory upregulation of other DUSPs, thereby reducing p38 activity.

## Conclusion

In conclusion, we found that ING4 significantly promoted the stemness enrichment of RCC cells by the p38 MAPK/type I IFN-stimulated gene signaling pathway. And ING4 regulated the activity of p38 MAPK by inhibiting the DUSP4 expression. We proposed that ING4 might serve as a potential therapeutic target for metastatic RCC, especially for RCC CSCs. Therefore, our deeper understanding of the role of ING4 in RCC will translate into a better therapeutic strategy for metastatic RCC, with overall higher success rates.

## Data Availability

The datasets presented in this study can be found in online repositories. The names of the repository/repositories and accession number(s) can be found at: https://www.ncbi.nlm.nih.gov; PRJNA799069.

## Abbreviations

RCC, renal cell carcinoma; ING4, inhibitor of growth 4; CSCs, cancer stem cells; IFN, type I interferon; ISGs, IFN-stimulated genes; DUSPs, dual-specificity phosphatases; MKPs, mitogen-activated protein kinase phosphatases; KIRC, renal clear-cell carcinoma; KIRP, renal papillary-cell carcinoma.
