# Supplementary material for: ING4 Promotes Stemness Enrichment of Human Renal Cell Carcinoma Cells Through Inhibiting DUSP4 Expression to Activate the p38 MAPK/type I IFN-Stimulated Gene Signaling Pathway
Source: Front Pharmacol. 2022 Apr 14;13:845097. doi: 10.3389/fphar.2022.845097 (PMC9046557; doi:10.3389/fphar.2022.845097)
Supplement: Supplementary file 1 [file DataSheet1.doc]

**Supplementary figures**

**
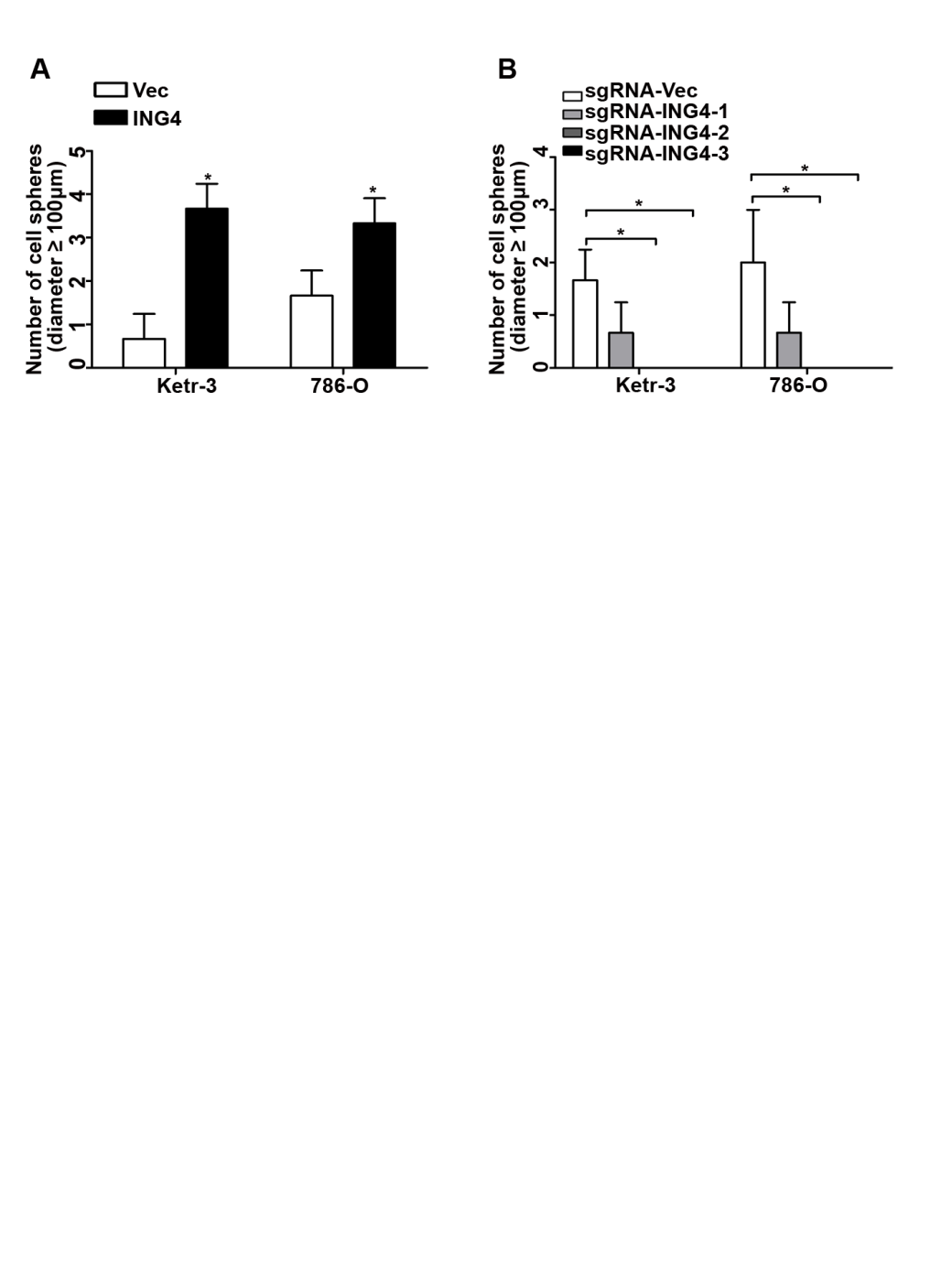
**

**Figure S1: ING4 increased the size of RCC cell sphere.** (A) The number of cell spheres with diameters ≥ 100μm in ING4 over-expression (ING4) and control (Vec) Ketr-3 and 786-O cells (n = 3). (B) The number of cell spheres with diameters ≥ 100μm in ING4 knockdown and control Ketr-3 and 786-O cells (n = 3). Data are presented as means ± standard deviation. *^*^P* < 0.05, *^**^P* < 0.001.

**
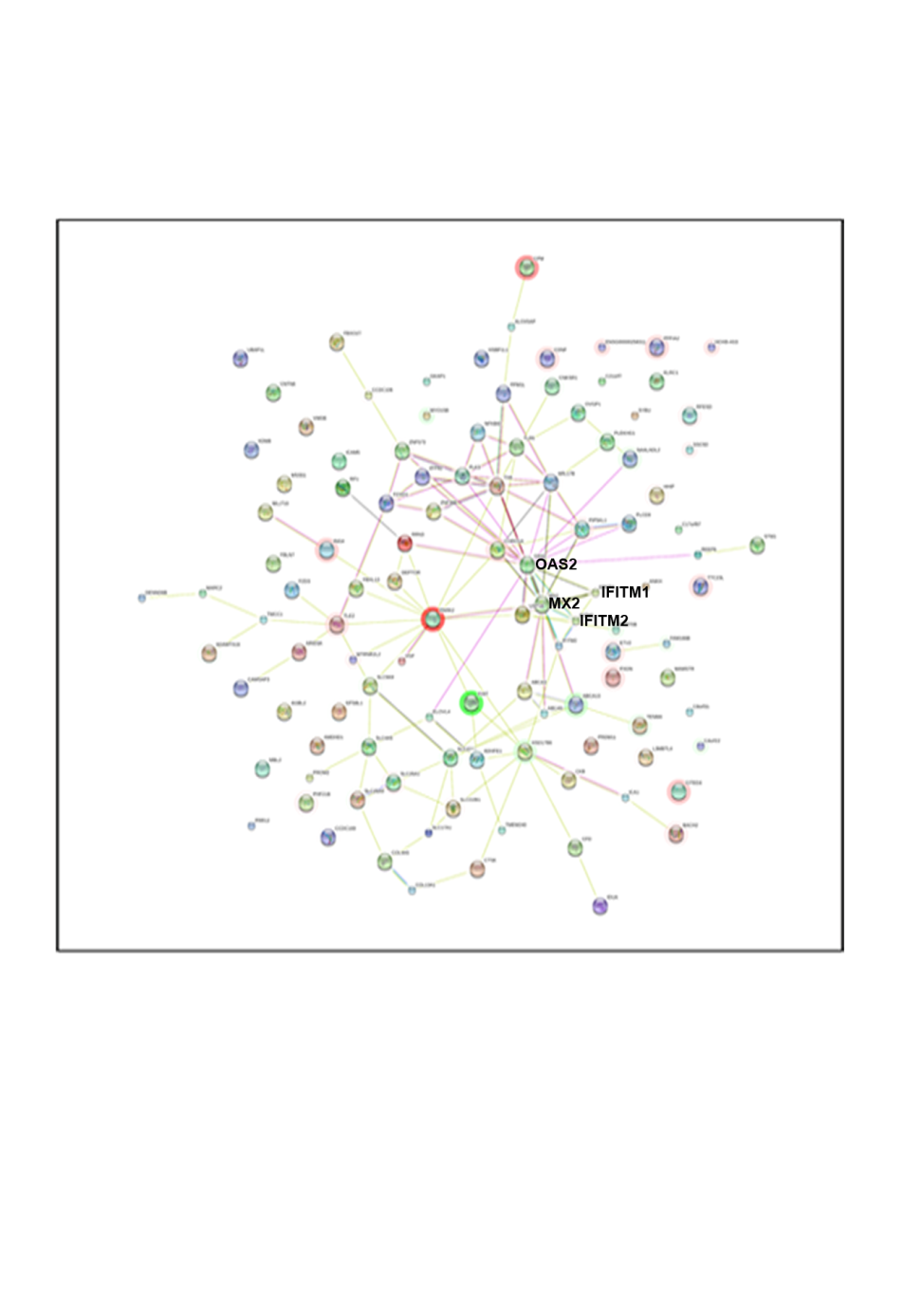
**

**Figure S2: IFN-stimulated genes (ISGs) IFITM1, IFITM2, MX2 and OAS2 were associated with many proteins and formed a core of protein interactions.** The protein interaction network showed that 400 associated proteins, 128 associated protein interactions, and 98 expected protein interactions in 786-O cells with stable ING4 over-expression compared to vector controls.

**
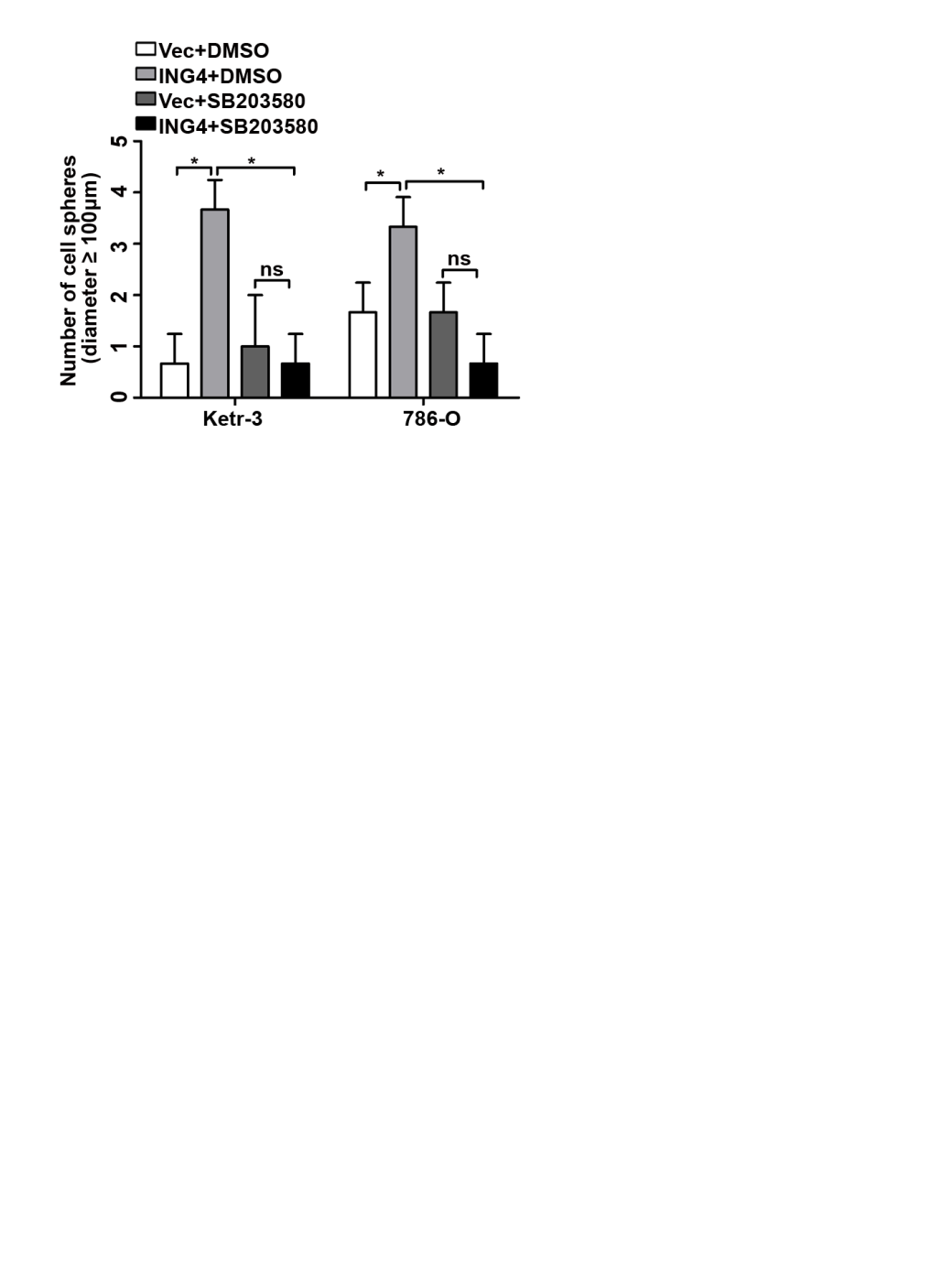
**

**Figure S3: SB203580 significantly inhibited the size of cell spheres promoted by ING4.** (A) The number of cell spheres with diameters ≥ 100μm in ING4 over-expression (ING4) or vector control (Vec) after 0.1%DSMO or 5μM SB203580 pretreatment for 24h (n = 3). Data are presented as means ± standard deviation. *^*^P* < 0.05, ns: no significance.


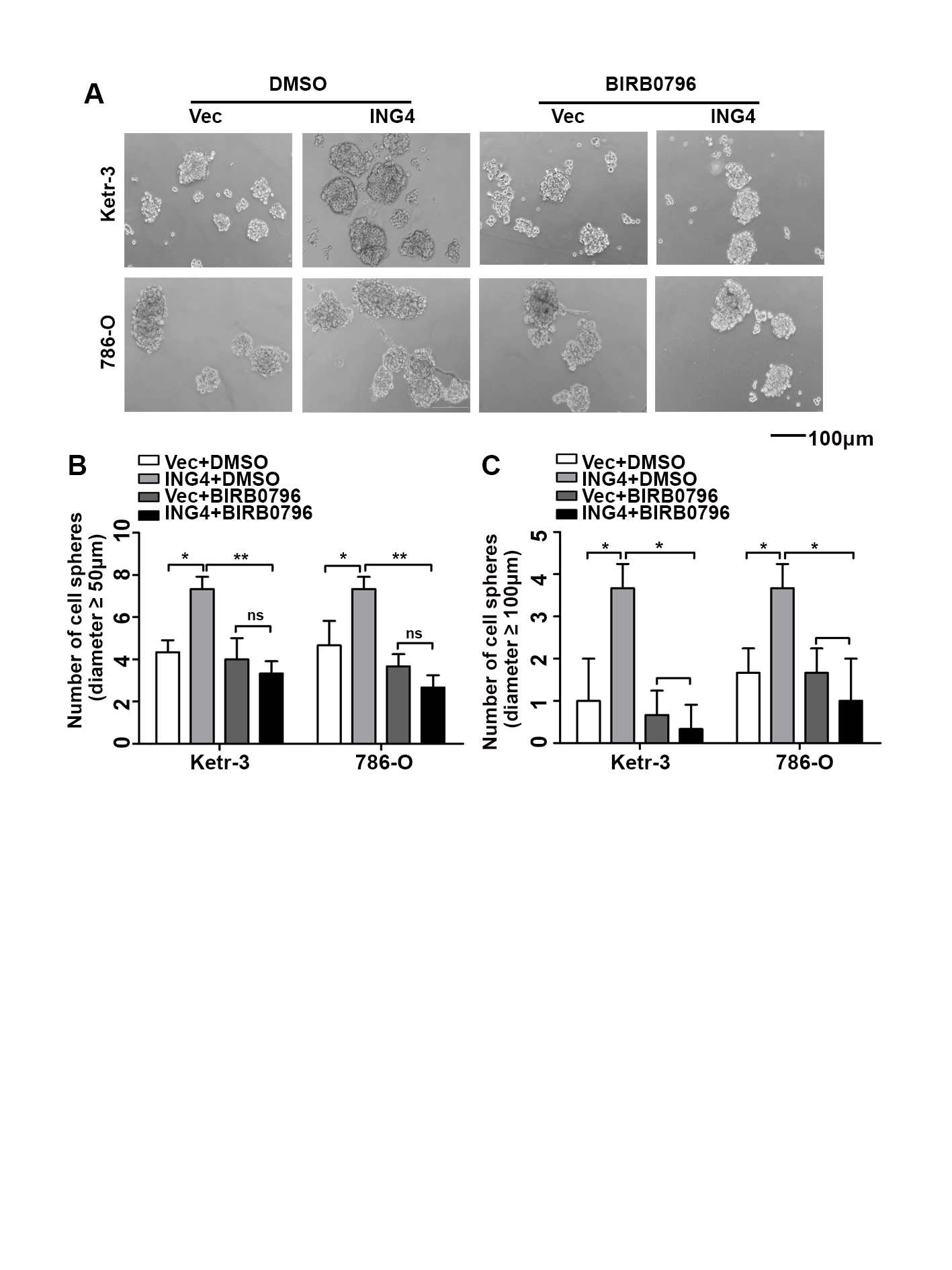


**Figure S4: BIRB0796 significantly inhibited cell sphere formation promoted by ING4.** (A) Photographs of cell spheres in Ketr-3 and 786-O cells with ING4 over-expression (ING4) or vector control (Vec) after 0.1%DSMO or 1μM BIRB0796 pretreatment for 24h (n = 3). (B-C) The number of cell spheres with diameters ≥ 50μm or ≥ 100μm. Note: images magnification, ×100; Scale bar, 100μm; Data are presented as means ± standard deviation. ^*^ *P*< 0.05, ^**^*P* < 0.001, ns: no significance.

**
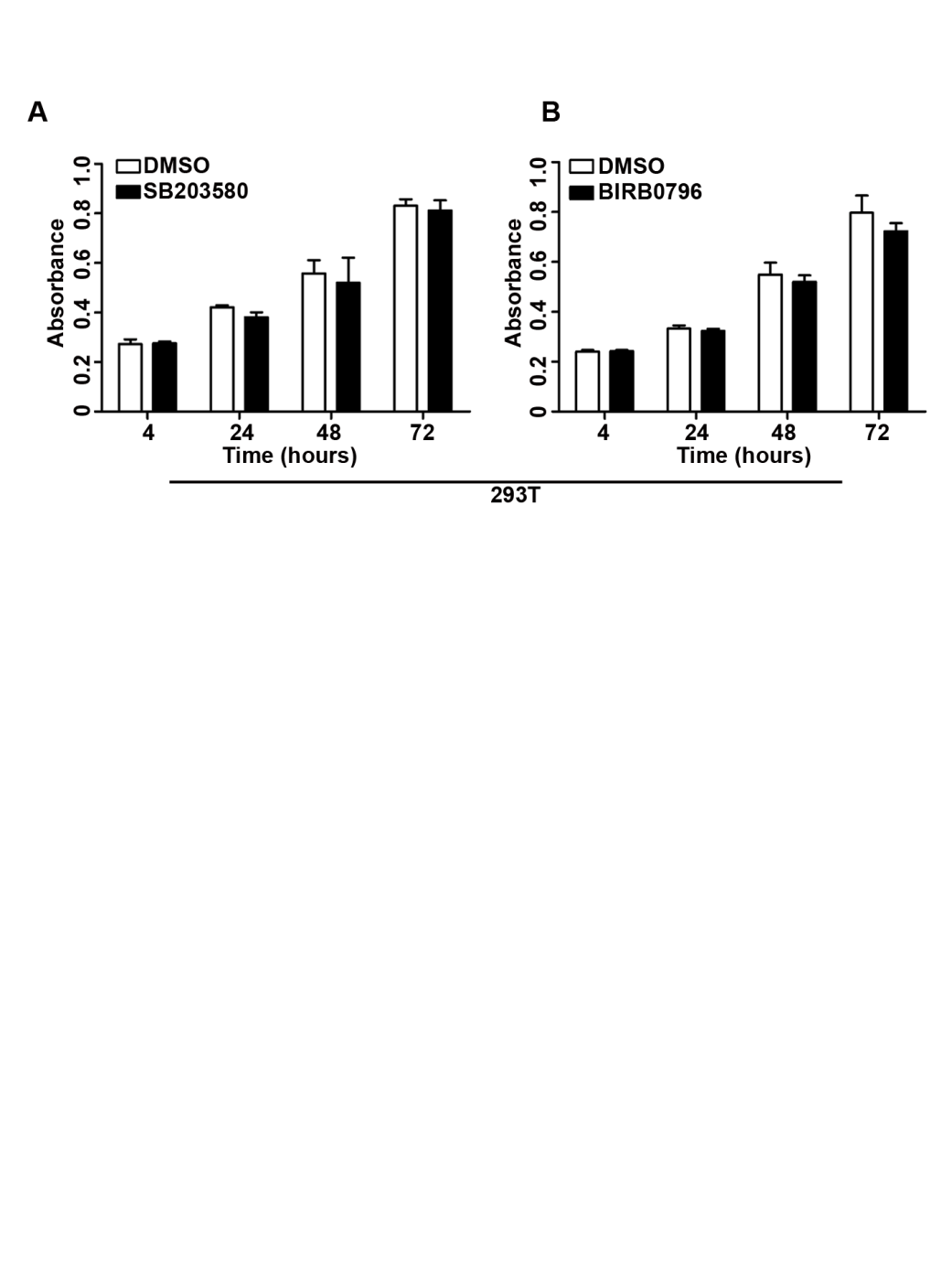
**

**Figure S5: p38 inhibitors had no significant inhibitory effect on the viability of normal human embryonic kidney cells 293T.** (A) The absorbance of 293T cells after treatment with SB203580, BIRB0796 or 0.1% DMSO for 4, 24, 48 and 72h. Data were presented as means ± standard deviations.

**Table S1** Relative expression levels of IFN-response genes in ING4 over-expressed 786-O cells (OE) compared to the controls (ctrl) in the transcriptome sequencing

| **GeneName** | **log2(OE/ctrl)** | **up-or-down** | ***P* value** |
| --- | --- | --- | --- |
| OAS2 | 0.733021 | up | 0.0011 |
| MX2 | 0.586243 | up | 0.00425 |
| IFITM1 | 0.90696 | up | 0.01465 |
| IFITM2 | 0.888416 | up | 5.00E-05 |
